# Docosahexaenoic acid supplementation in phenylketonuria: a systematic review

**DOI:** 10.3389/fnut.2025.1676666

**Published:** 2025-10-10

**Authors:** Manuel Alanís-Bernal, Laia Boada-Oller, Mihir Sojitra

**Affiliations:** ^1^Neurology Department, Vall d'Hebron University Hospital, Barcelona, Spain; ^2^Independent Researcher, Formerly at B. J. Medical College, Gujarat University, Ahmedabad, India

**Keywords:** systematic review, phenylketonuria (PKU), docosahexaenoic acid (DHA), long-chain polyunsaturated fatty acids (LC-PUFA), neurodevelopment, nutrition, dietary supplementation

## Abstract

**Background:**

Phenylketonuria (PKU) is an inborn error of metabolism requiring a protein-restricted diet, which may predispose patients to deficiencies in long-chain polyunsaturated fatty acids (LC-PUFAs), particularly docosahexaenoic acid (DHA). Given the critical role of DHA in neurodevelopment and membrane function, supplementation has been proposed as a potential therapeutic adjunct in PKU. To systematically review clinical and biochemical outcomes associated with DHA supplementation in individuals with PKU.

**Methods:**

A systematic literature search was conducted in PubMed for studies published between January 1990 and July 2025. We included clinical trials, cohort studies, and observational studies assessing the effects of DHA or LC-PUFA supplementation in PKU. Data were extracted regarding study design, sample size, age, dosage, duration, and outcome measures. Risk of bias was assessed using the Cochrane tool and ROBINS-I as appropriate.

**Results:**

A total of 18 studies met the inclusion criteria. DHA supplementation consistently increased erythrocyte and plasma DHA levels, and some trials reported improvements in visual evoked potentials, while cognitive outcomes remained inconsistent. No adverse effects were reported.

**Conclusion:**

DHA supplementation appears to be a safe and effective strategy to correct LC-PUFA deficiency in PKU, with potential neurophysiological benefits. However, its impact on long-term neurocognitive development remains uncertain, and further high-quality studies are needed to clarify its clinical value.

## Introduction

Phenylketonuria (PKU) is an autosomal recessive inherited metabolic disorder caused by a deficiency of the enzyme phenylalanine hydroxylase, responsible for the conversion of phenylalanine into tyrosine (1). Treatment relies on a strict low-phenylalanine diet supplemented with specialized amino acid mixtures to ensure adequate protein intake without toxic accumulation of phenylalanine (2, 3). Although this intervention significantly improves neurological outcomes, it can lead to secondary nutritional deficiencies, including a reduced intake of long-chain polyunsaturated fatty acids (LCPUFAs), particularly docosahexaenoic acid (DHA), which is essential for central nervous system development (2–4).

PKU is rare but not negligible. A recent systematic analysis estimated its global prevalence at approximately 1 in 23,930 newborns, with considerable regional variation, including approximately 1 in 10,000 in Europe and around 1 in 5,500 in Turkey (5). These updated data emphasize that, despite being an uncommon condition, PKU continues to represent a relevant public health issue (6, 7).

DHA is a major structural component of neuronal and retinal membranes. Several studies have reported decreased levels of DHA in the plasma and erythrocytes of patients with PKU (8–10), which has prompted investigations into the potential benefits of DHA supplementation. The results so far have been heterogeneous: while some trials have shown improvements in visual evoked potentials or neurocognitive development, others have failed to demonstrate significant clinical effects.

Therefore, the objective of this systematic review is to examine the scientific literature published up to 2025 regarding DHA supplementation in patients with phenylketonuria, assessing both biochemical and functional outcomes, with the aim of evaluating the consistency of the findings and their potential clinical implications.

## Materials and methods

This systematic review was conducted in accordance with the PRISMA 2020 guidelines (11).

### Eligibility criteria

We included studies that assessed the effects of DHA supplementation—either alone or in combination with other LC-PUFAs—in patients diagnosed with PKU. Eligible studies were required to involve human participants, be published in English or Spanish, include an available abstract, and use one of the following designs: randomized controlled trials, non-randomized interventional studies, prospective or cross-sectional observational studies, or systematic reviews of such designs. Conference abstracts were excluded because they are not peer-reviewed, typically lack sufficient methodological detail, and do not allow reliable assessment of outcomes or study quality.

Studies were excluded if they did not fulfill the objective of this review—namely, reporting at least one outcome related to DHA status (clinical, neurocognitive, neurophysiological, or biochemical)—or if they did not specifically investigate DHA supplementation in the target populations.

### Information sources

A single electronic database—PubMed—was used for the literature search. Reference lists of the included articles were also screened to identify any additional relevant publications.

Although additional databases such as Embase or Scopus may contain relevant studies, PubMed was selected due to its comprehensive indexing of biomedical literature and its focus on peer-reviewed, clinically relevant publications. Given the specificity of the topic and the high-quality sources indexed, we considered it sufficient for the scope of this systematic review.

### Search strategy

The search was conducted in PubMed to identify studies published between January 1990 and July 2025. A combination of Medical Subject Headings (MeSH) and free-text keywords was used: “docosahexaenoic,” “arachidonic,” “LC-PUFAs,” “PUFAs,” “phenylketonuria,” and “PKU.” Boolean operators “AND” and “OR” were used to refine the search.

The final search query was as follows: (LC-PUFAs OR PUFAs OR ARACHIDONIC OR DOCOSAHEXAENOIC) AND (PHENYLKETONURIA OR PKU).

### Study selection

The initial search retrieved 141 articles. After screening titles and abstracts according to the eligibility criteria, 30 studies were selected for full-text review. Twelve of these were excluded due to reasons such as inclusion of healthy individuals (*n* = 2), lack of relevance to PKU (*n* = 5), or other reasons (*n* = 4), such as failure to meet the study objective, insufficient information on DHA supplementation, or methodological limitations preventing data extraction. A total of 18 studies met all inclusion criteria and were incorporated into the final analysis.

**Figure 1:**
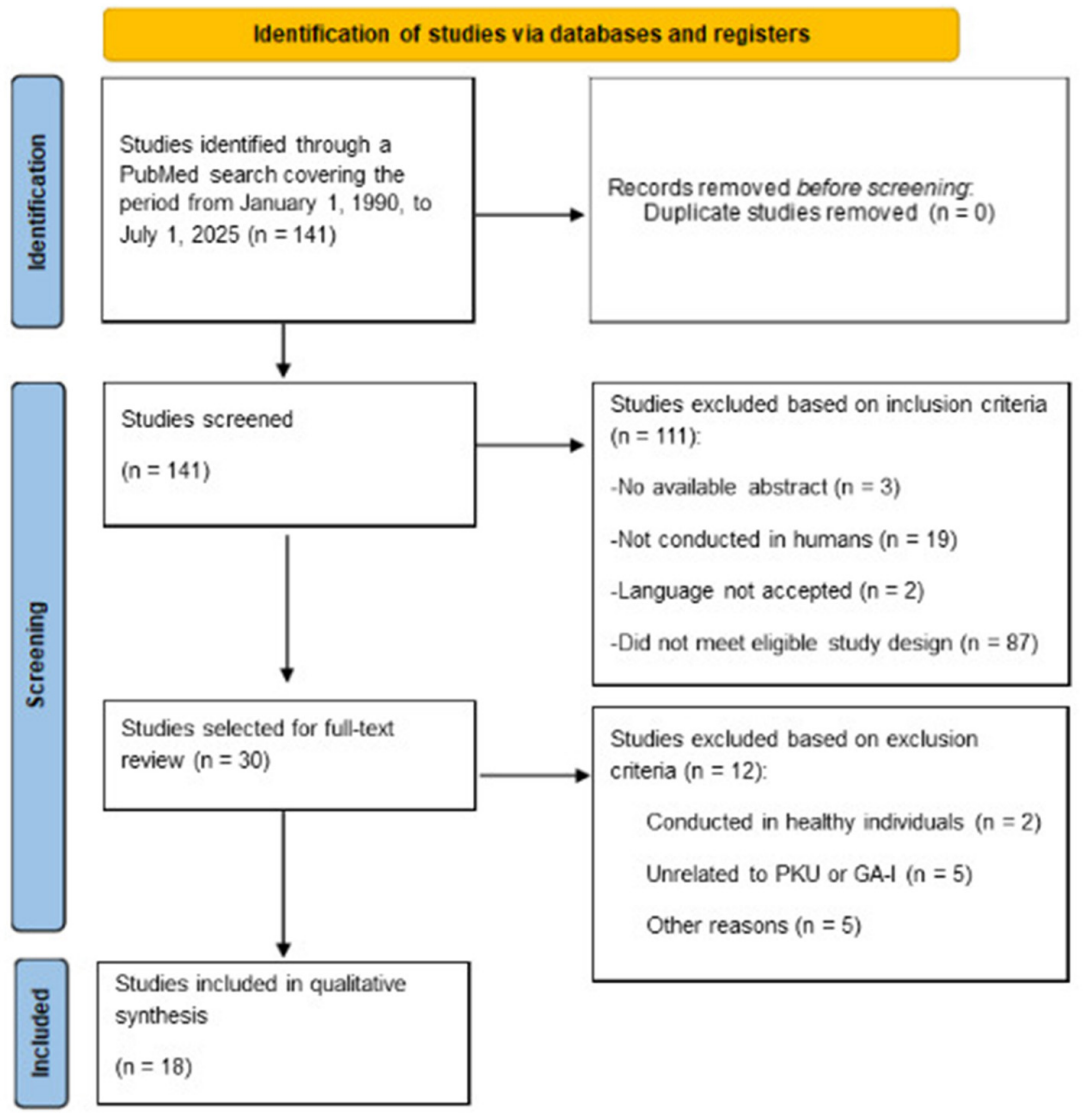
PRISMA 2020 flow diagram of the study selection process. PKU, Phenylketonuria.

The study selection process is illustrated in the PRISMA flow diagram (Figure 1).

### Data extraction and synthesis

From each included study, we extracted the following information: year of publication, study design, number of participants, age, diagnosis, DHA dosage and duration, outcome measures, and main findings. The data were summarized in a structured table for descriptive analysis. Given the clinical and methodological heterogeneity across studies, no meta-analysis was performed.

For systematic reviews, narrative reviews and meta-analyses, no primary data (e.g., sample size, age, DHA dose, and duration) were extracted, and these studies are only described qualitatively.

### Risk of bias assessment

The risk of bias for each included study was independently assessed by two reviewers. For randomized controlled trials, the Cochrane Risk of Bias 2.0 tool was applied; for non-randomized and observational studies, the ROBINS-I tool was used. Any discrepancies in judgment were resolved through discussion and consensus.

## Results

### Characteristics of included studies

A total of 18 studies met the inclusion criteria. Eighteen intervention studies were conducted in patients with PKU. The intervention studies encompassed randomized controlled trials, open-label interventions and observational designs. Most focused on pediatric patients, though some included adolescents or adults. When DHA supplementation was given, doses ranged from 5–15 mg/kg/day or fixed daily amounts and were administered for 3 to 12 months; several of these trials combined DHA with arachidonic acid (AA) (12–29). All included studies are summarized in Table 1.

**Table 1:** Summary of studies on long-chain polyunsaturated fatty acids in phenylketonuria.

**Author (Year) [Ref]**	**Study design**	**Sample size (n)**	**Age range**	**DHA dose & duration**	**Outcomes evaluated**	**Main findings**
Beblo et al. (2001) (12)	Prospective controlled trial with fish-oil supplementation	36 children with PKU	1–11 y	15 mg DHA/kg/day + 22.5 mg EPA/kg/day for 3 months	Erythrocyte fatty acid composition; visual evoked potential (P100 latency)	Increased DHA levels and reduced P100 latency, improving visual function
Agostoni et al. (2003) (13)	Double-blind randomized trial with 3-yr follow-up	20 hyperphenylalaninaemic children	~10 y	10 mg DHA/kg/day + 8 mg EPA/kg/day for 12 months	Plasma DHA and P100 latency	Improvements at 12 months but no differences at 3 years
Agostoni et al. (1995) (14)	Double-blind placebo-controlled trial	21 children with HPA	5–10 y	15 mg DHA/kg/day + 22.5 mg EPA/kg/day for 6 months	Lipid profile and plasma fatty acids	Decrease in triglycerides and increase in DHA/EPA without adverse effects
Gutiérrez-Mata et al. (2012) (15)	Prospective one-year study	21 PKU patients	9–25 y	10 mg DHA/kg/day for 12 months	Biochemical parameters, MRI, visual evoked potentials, neuropsychological tests	Supplementation normalized DHA; P100 latency decreased and fine motor skills improved; no change in other functions
Koletzko et al. (2007) (16)	Randomized controlled trial	54 children (PKU and controls)	Mean 6.3 ± 0.6 y	15 mg DHA/kg/day + 22.5 mg EPA/kg/day for 3 months	Fine motor skills and balance	Supplemented group showed significant improvements compared with healthy controls
Agostoni et al. (2006) (17)	Double-blind randomized trial in infants	42 infants with PKU	~20 ± 6.9 wk	Phe-free formula with 0.3 g DHA/100 g + 0.7 g AA/100 g for 12 months	Mental/psychomotor development, DHA levels, P100 latency	Supplement prevented decline in DHA; no differences in mental development or P100 latency
Agostoni et al. (2001) (18)	Double-blind trial with placebo	20 school-age HPA children	≈ 6–11 y	Capsules containing 26 % long-chain PUFA (0.3–0.5 % of energy, 8 % DHA) for 12 months	DHA in plasma phospholipids and lipids	Supplementation doubled plasma DHA without altering arachidonic acid or lipid profile
Rose et al. (2005) (19)	Randomized trial in PKU children	43 children (24 intervention; 19 control)	2–12 y	Phe-free protein substitute enriched with essential fatty acids for 20 weeks	Fat and DHA intake, nutritional status	Intervention group had higher fat and DHA intake; younger children on fat-free formula had fat deficits
Cleary et al. (2006) (20)	Randomized controlled trial	53 children with PKU	1–10 y	Formula with essential fatty acids for 20 weeks	Erythrocyte DHA and AA	Erythrocyte DHA increased 19 % vs 0.5 % in controls; AA unchanged
Yi et al. (2011) (21)	Double-blind placebo-controlled pilot trial	33 females with PKU	12–47 y	10 mg DHA/kg/day for 4.5 months	Cognitive processing speed and executive function	DHA biomarkers increased; no differences in cognitive performance between DHA and placebo
Infante and Huszagh (2001) (22)	Mechanistic review article	N/A	N/A	N/A	Synthesis of arachidonic acid and DHA; effects of phenylalanine metabolites	Suggests phenylalanine metabolites inhibit AA and DHA synthesis; proposes supplementation with carnitine, AA and DHA
Giovannini et al. (1995) (23)	Narrative review	N/A	N/A	N/A	Lipid status and fatty acid metabolism in PKU	Highlights that low consumption of animal products leads to low cholesterol and long-chain PUFA; recommends LCPUFA supplementation in PKU infants
Ryan et al. (2010) (24)	Review of human studies	N/A	N/A	N/A	Neurodevelopment and long-chain PUFA	Concludes that DHA/EPA supplementation improves visual and motor functions in children and is well tolerated
Lohner et al. (2013) (25)	Systematic review and meta-analysis	9 case-control studies and 6 RCTs	N/A	N/A	DHA/EPA in biomarkers	PKU patients have significantly lower DHA/EPA; supplementation increases plasma DHA; functional benefits remain uncertain
Giovannini et al. (2007) (26)	Review of dietary and therapeutic challenges	N/A	N/A	N/A	Dietary and therapeutic aspects of PKU	Emphasizes the need for permanent LCPUFA supplementation for CNS development and for preventing micronutrient deficiencies
Stroup et al. (2018) (27)	Metabolomic cross-over/observational study	25 adults with PKU	18–49 y	Habitual diet; fatty acid and metabolite profiles analyzed	n-6:n-3 ratio, DHA/EPA, carnitine and metabolites	Found elevated n-6:n-3 ratio and low DHA/EPA with altered carnitine; conclude that DHA supplementation and improved carnitine bioavailability are needed
Feillet and Agostoni (2010) (28)	Review of nutritional issues	N/A	N/A	N/A	Phe-restricted diet	Notes that the PKU diet resembles a vegan diet; stresses the importance of high-quality amino acid formulas and adding LCPUFA and micronutrients to prevent deficiencies
Couce et al. (2019) (29)	Systematic review of controlled trials	12 controlled trials	N/A	≥ 10 mg DHA/kg/day (varied)	P100 latency and neurocognitive variables	Doses ≥ 10 mg/kg/day reduce P100 latency, but evidence for cognitive improvement is inconclusive; optimal dose not defined

The table includes randomized controlled trials, observational studies, and reviews reporting on DHA supplementation or fatty acid status. Data on sample size, age, DHA dose, and supplementation duration are only shown for original studies; reviews and meta-analyses are included for reference but these data are not applicable (N/A).

RCT, randomized controlled trial; VEP, visual evoked potentials; PKU, phenylketonuria; DHA, docosahexaenoic acid; EPA, eicosapentaenoic acid; AA, arachidonic acid; LCPUFA, long-chain polyunsaturated fatty acids; RBC, red blood cell; y, years; m, months.

### Fatty acid profile outcomes

Among the 18 studies, 14 reported erythrocyte or plasma DHA levels before and after supplementation. Most described a significant post-supplementation increase, confirming baseline deficiency and suggesting a biochemical response (12, 13, 15, 16, 18–20, 22–29). However, results were heterogeneous across studies, and long-term follow-up data were limited. In some trials, improvements were also seen in omega-6/omega-3 ratios or arachidonic acid levels, although findings were variable.

### Neurophysiological assessments

Five studies assessed visual evoked potentials (VEPs), focusing on P100 latency as a marker of neural conduction. A reduction in latency was observed in three of them (12, 19, 24), while two found no significant changes after supplementation (16, 17). Overall, the small number of studies and methodological differences prevent drawing firm conclusions regarding neurophysiological effects.

### Cognitive and neuropsychological outcomes

Five studies evaluated cognitive outcomes using standardized neuropsychological assessments (14, 16, 17, 22, 27). While some studies described minor trends toward improvement, none reported statistically significant changes attributable to DHA. Interpretation of these findings is substantially limited by the small sample sizes and the diversity of testing tools used.

### Safety and tolerability

None of the studies reported adverse effects related to DHA supplementation. Across all 18 studies, DHA was described as well tolerated, with no negative impact on laboratory parameters or clinical outcomes (12–29). Nevertheless, safety data are restricted by the relatively short follow-up periods and the limited number of participants.

## Discussion

A total of 18 studies were included in this systematic review. While most focused on the biochemical effects of DHA supplementation in patients with PKU, only a minority addressed functional outcomes, such as neurophysiological or cognitive performance.

This review confirms that DHA supplementation consistently increases blood DHA levels in individuals with PKU. However, the translation of these biochemical changes into clinically meaningful benefits remains unproven, as the few available trials on neurophysiological and cognitive outcomes did not demonstrate significant or reproducible improvements.

The homogeneity of the biochemical response is notable and expected, given the correction of a well-defined dietary deficiency. However, the clinical utility of this finding is questionable, and isolated improvements in erythrocyte or plasma DHA levels, in the absence of functional benefit, do not justify routine supplementation. The reduction in P100 latency seen in a few studies may hint at neural effects, but these results are inconsistent and based on small samples, limiting their reliability.

Cognitive assessments, although performed in several studies, failed to demonstrate consistent improvements following DHA supplementation. Recurrent methodological limitations—including small sample sizes, short supplementation periods, heterogeneous and non-standardized testing tools, and lack of blinding—further weaken the evidence. In addition, the literature search was limited to PubMed, and relevant studies indexed in other databases may have been missed. Another important limitation is the absence of recent interventional evidence: no clinical trials on DHA supplementation in PKU have been published between 2020 and 2025, underscoring a persistent gap in contemporary research. Moreover, the potential contribution of protein substitutes and medical foods, which frequently contain added DHA, should be acknowledged, as this may confound baseline deficiency assessments and the interpretation of supplementation effects. Finally, the absence of a meta-analysis or forest plots reflects the limited number and heterogeneity of studies, and further restricts the statistical strength of the conclusions.

While most of the included studies were small and heterogeneous, none reported adverse effects, suggesting that DHA supplementation is safe and well tolerated. Nevertheless, the limited follow-up and the low number of participants prevent definitive conclusions on long-term safety. Safety alone cannot be considered sufficient justification for widespread use in the absence of proven efficacy. Future studies should not only report biochemical changes but should also be powered to detect functional outcomes, with pre-specified endpoints, appropriate controls, and longer follow-up.

In summary, DHA supplementation effectively corrects biochemical DHA deficiency in patients with PKU, but current evidence does not support clinically relevant benefits on neurocognitive or neurophysiological outcomes. Routine supplementation in PKU should not be recommended; instead, decisions should be individualized and research efforts prioritized to clarify its true clinical value.

## Conclusion

This systematic review demonstrates that DHA supplementation reliably increases circulating DHA levels in individuals with PKU, confirming its biochemical efficacy. However, current evidence does not support a consistent improvement in neurocognitive or neurophysiological outcomes, limiting its clinical utility. Given the lack of demonstrated functional benefits, the routine use of DHA supplementation in PKU should not be recommended; instead, decisions should be individualized, weighing potential biochemical correction against uncertain clinical impact. Future high-quality, adequately powered trials are essential to determine whether DHA can offer meaningful therapeutic benefits beyond biochemical normalization.

## Data Availability

The original contributions presented in the study are included in the article/supplementary material, further inquiries can be directed to the corresponding author.
